# Food Safety and Nutraceutical Potential of Caramel Colour Class IV Using In Vivo and In Vitro Assays

**DOI:** 10.3390/foods8090392

**Published:** 2019-09-05

**Authors:** Marcos Mateo-Fernández, Pilar Alves-Martínez, Mercedes Del Río-Celestino, Rafael Font, Tania Merinas-Amo, Ángeles Alonso-Moraga

**Affiliations:** 1 Department of Genetic, Rabanales Campus, University of Córdoba (UCO), 14071 Córdoba, Spain; 2 Agri-Food Laboratory, Council of Agriculture, Fisheries and Rural Development of Andalusia (CAPDER), 14004 Córdoba, Spain

**Keywords:** caramel colour E150d-Class IV (CAR), nutraceutical potential, food safety

## Abstract

Nutraceutical activity of food is analysed to promote the healthy characteristics of diet where additives are highly used. Caramel is one of the most worldwide consumed additives and it is produced by heating natural carbohydrates. The aim of this study was to evaluate the food safety and the possible nutraceutical potential of caramel colour class IV (CAR). For this purpose, in vivo toxicity/antitoxicity, genotoxicity/antigenotoxicity and longevity assays were performed using the *Drosophila melanogaster* model. In addition, cytotoxicity, internucleosomal DNA fragmentation, single cell gel electrophoresis and methylation status assays were conducted in the in vitro HL-60 human leukaemia cell line. Our results reported that CAR was neither toxic nor genotoxic and showed antigenotoxic effects in *Drosophila.* Furthermore, CAR induced cytotoxicity and hipomethylated sat-α repetitive element using HL-60 cell line. In conclusion, the food safety of CAR was demonstrated, since Lethal Dose 50 (LD_50_) was not reached in toxicity assay and any of the tested concentrations induced mutation rates higher than that of the concurrent control in *D. melanogaster*. On the other hand, CAR protected DNA from oxidative stress provided by hydrogen peroxide in *Drosophila*. Moreover, CAR showed chemopreventive activity and modified the methylation status of HL-60 cell line. Nevertheless, much more information about the mechanisms of gene therapies related to epigenetic modulation by food is necessary.

## 1. Introduction

From the beginnings, humanity has been searching for different methods in order to improve our feeding. Adding molecules to increase the flavour or to get a better preservation of food is one of these methods. Its consequence is the appearance of a problem: the quality of food which has been altered with additives. According to the “Codex Alimentary”, an additive is “any substance not normally consumed as a food by itself and not normally used as a typical ingredient of the food, whether or not it has nutritive value, the intentional addition of which to food for a technological (including organoleptic) purpose in the manufacture, processing, preparation, treatment, packing, transport or holding of such food results, or may be reasonably expected to result (directly or indirectly), in it or its by-products becoming a component of or otherwise affecting the characteristics of such foods. The term does not include contaminants or substances added to food for maintaining or improving nutritional qualities”.

Natural food additives, such as salt, vinegar, wine and spices have been extensively used in order to preserve foods and improve their organoleptic properties. However, synthetic additives are the most used nowadays. The food additives consumption is regulated for maintaining the quality and health security of food. New additives must undergo toxicity and carcinogenesis studies before entering in the market. These in vivo assays are performed in order to determine the carcinogenic potential of these compounds. This is the reason why some new additives are safer than other compounds used for years. With this in mind, coal tar has been used since 1956, although carcinogenic risks for the consumers were noticed only when researchers began studying it in in vivo assays [1], and more recently, studies have showed that some molecules incorporated in food could cause cancer [2]. For this reason, a food safety evaluation is needed [3].

It is known that dietary compounds are related to the induction or prevention of several diseases. As a proof of that, patients from developing and underdeveloped countries suffer from different kinds of cancer that could be related to the increase of the food additives consumption [1]. Therefore, the relationship between diet and health is very close [4], and food genotoxicologic assays have been widely used through time in order to evaluate the healthy properties of diet before being considered as nutraceutic substances [5,6]. Nowadays, there is a great deal of scientific evidence based on nutraceuticals, supporting the idea that a deliberate consume of certain food can be a health promoter.

Caramel is one of the most worldwide consumed additives and is produced by heating carbohydrates from vegetable sources (glucose, sucrose, invert sugar, etc.) in the presence of caramelisation promoters (ammonia or ammonium in class III and IV, respectively). The result is a complex mixture which is responsible for the aromatic and colourant characters of caramels [7,8], and it has been used in food and beverages to provide some properties such as colour, taste, smell and texture, and for its ability for stabilisation of colloidal systems, as well as for its emulsifying properties, facilitating the dispersion of water-insoluble materials, retarding flavour changes and preserving the shelf-life of beverages exposed to light [9]. Besides these, caramel has recently also been highlighted as beneficial in nonenzymatic browning inhibition [10].

*Drosophila* is a reliable model to evaluate the toxicity, genotoxicity and other degenerative processes of food or chemical structures [11]. The results obtained in this eukaryote organism are considered translational and highly specific, as more than 80% of genes related to human disease are homologous in *Drosophila* [12]. The SMART (Somatic Mutation and Recombination Test) is a useful tool for in vivo genetic studies with *D*. *melanogaster*. The SMART is based on the genetic alterations produced in the cells of imaginal discs of larvae. These alterations can phenotypically be expressed in adult tissues after clonal expansion and metamorphosis. The SMART has shown to be able to detect genotoxic activity of various compounds with different chemical structures, either as direct mutagens or promutagens, and with different genotoxic action modes, such as alkylating, intercalating agents, adducts formers [11] as well as complex mixtures [13,14,15]. The detection of genotoxic and antigenotoxic agents is important since they can be considered as carcinogenic or anticarcinogenic substances, respectively.

In addition, *Drosophila melanogaster* is an excellent model for the study of aging, because adults show many similarities with the cellular senescence observed in mammals [16]. This is the reason why this particular model is frequently used to understand the relationship between nutrient metabolism and aging mechanisms [17], and further substantial contributions in this sense are expected [18].

In parallel, in vitro cytotoxicity assays are used to assess the chemopreventive potential of compounds [19]. Taking into account that cancer therapies are highly toxic and mainly unspecific, an alternative strategy could be the use of agents able to induce cell differentiation in cancerous cells [20]. HL-60 human leukaemia cell line is widely used to detect the capability of inducing cell differentiation and proapoptotic mechanisms of the compounds to be tested. These compounds could be considered as chemopreventive agents [21,22].

DNA methylation is an epigenetic mark that shows the transcriptional gene silencing and that plays a vital role during development and in the genome defence against transposable elements [23]. The methylation status of these transposable sequences is relevant for understanding the global DNA methylation. Prevention or reversal of hypermethylation-induced inactivation of tumour suppression genes or gene receptors by DNA Methyltransferase (DNMT) inhibitors could be an effective approach to cancer prevention [24].

Taking that into account, environmental exposures to nutritional, chemical and physical factors (such as smoke, diet and physical activity) could alter human and animal gene expression and modify the susceptibility to disease due to epigenomic changes [25]. Currently, biomedical research is focused on modifying the methylation pattern as a tool to understand cancer processes and others diseases. The ability of food compounds to influence the epigenome in cancer cells has been studied and has also been related to the individual’s risk of developing cancer. Since epigenetic changes can be reversed in the human lifespan, the epigenetic focus is a good tool for the dietary prevention/treatment of cancers.

In vivo toxicity, antitoxicity, genotoxicity, antigenotoxiciy and lifespan assays using the model organism *Drosophila melanogaster*, and in vitro cytotoxicity, DNA fragmentation, methylation status and comet assays using HL-60 promyelocytic human cell line were carried out to evaluate some biological activities related to degenerative processes, food security and nutraceutic potential of CAR (caramel colour E150d-class IV).

## 2. Materials and Methods

### 2.1. Samples

A colour additive, caramel colour E150d-class IV (CAR), was selected for this study and was kindly provided by SANCOLOR (Barcelona, Spain).

### 2.2. In Vivo Fly Stocks

Two *Drosophila melanogaster* strains with genetic markers that affect the wing-hair phenotype were used: (i) *mwh/mwh*, carrying the recessive mutation *mwh* (multiple wing hairs) [26] and (ii) *flr3/In (3LR) TM3, rip^p^se^p^ bx^34e^e^s^Bd^S^*, where *flr^3^* (*flare*) [27] marker is a homozygous recessive lethal mutation which is viable in homozygous somatic cells once larvae start developing and produce deformed trichomonas.

### 2.3. In Vitro Cell Culture Conditions

Promyelocytic human leukaemia (HL-60) cells were grown in RPMI-1640 medium (R5886, Sigma-Aldrich, St. Louis, MO, USA) supplemented with heat-inactivated foetal bovine serum (Linus, S01805, Madrid, Spain), L-glutamine 200 mM (G7513, Sigma-Aldrich, St. Louis, MO, USA) and 1× antibiotic–antimycotic solution (A5955, Sigma-Aldrich, St. Louis, MO, USA). Cells were incubated at 37 °C in a humidified atmosphere of 5% CO_2_. Cultures were plated at 2.5 × 10^4^ cells/mL density in 10 mL culture bottles and passed every 2 days.

### 2.4. In Vivo Safety Studies

#### 2.4.1. Toxicity Assays

Toxicity was assayed according to our standard protocols. Before carrying out the assays, colour caramel was dissolved in distilled water in order to obtain the different concentrations as follows: 0.03, 0.125, 0.25, 1 and 4 mg/mL for CAR. The CAR concentrations range was calculated in order to assay the same amounts that are contained in the cola beverages [28]. A negative (H_2_O) concurrent control was also assayed. Test groups consisted of larvae fed with *Drosophila* Instant Medium (Formula 4-24, Carolina Biological Supply, Burlington, NC, USA) supplemented with the tested compounds concentrations. Emerging adults of all groups were counted, and toxicity was determined as the percentage of hatched individuals in each treatment compared with the negative control.

Chi-square test was used to determine if the tested compounds significantly affected the survival of flies, as it was previously described by Mateo-Fernández, et al. [15], where an in parallel lethal dose 50 (LD_50_) was performed to ascertain the toxicity of the compounds.

#### 2.4.2. Genotoxicity Assay

Genotoxicity assays were carried out following the wing spot test standard procedure [11]. Briefly, transheterozygous larvae for *mwh* and *flr^3^* genes were obtained by crossing four-day-old virgin *flr^3^* females with *mwh* males in a 2:1 ratio. Four days after fertilization, females were allowed to lay eggs in fresh yeast medium (25 g yeast and 4mL sterile distilled water) for 8 h in order to obtain synchronised larvae. After 72 h, larvae were collected, washed with distilled water and clustered in groups of 100 individuals. Each group was fed with a mixture containing 0.85 g *Drosophila* Instant Medium (Formula 4-24, Carolina Biological Supply, Burlington, NC, USA) and 4 mL water, supplemented with the tested compounds at fixed concentrations (the highest and second lowest from the toxicity assays) and negative (H_2_O) and positive (0.15 M H_2_O_2_) controls, until pupae hatching (10–12 days). Adult flies were collected and mounted on slides using Faure’s solution. Mutant spots were scored in both dorsal and ventral surfaces of the wings in a bright light microscope at 400× magnification.

The frequencies of each type of mutant clone per wing (single, large or twin spot) were compared to the concurrent negative control and analysed by applying the Kastenbaum and Bowman binomial test [29]. Inconclusive results from this binomial test were resolved by using Mann–Whitney and Wilcoxon U-test [14,30].

### 2.5. In Vivo Evaluation of Nutraceutical Potential

#### 2.5.1. Antitoxicity Assay

Antitoxicity was assessed using the same procedure and experimental concentrations as in toxicity assays, but in combined treatments with 0.15 M H_2_O_2_ and comparing the percentage of emerging adults with the positive toxicant control [31]. Chi-square test was also used for comparing treatments to the positive concurrent control [15].

#### 2.5.2. Antigenotoxicity Assay

Antigenotoxicity tests were performed, following the method described by Anter, et al. [13]. The same concentrations used in genotoxicity assay were assayed in combined treatment with hydrogen peroxide (0.15 M) acting as concurrent genotoxicant. The inhibition percentages (IP) for the combined treatments were calculated when appropriate according to Abraham and Singh [32] formula:

IP = ((genotoxin alone − combined treatment)/genotoxin alone) × 100

Inconclusive results from Kastembaum–Bowman binomial test were also resolved using Mann–Whitney U-test [14,30].

#### 2.5.3. Chronic Treatments: Lifespan and Healthspan Assays

In order to obtain comparable results in all the in vivo assays, we used an F_1_ progeny from *mwh* and *flr^3^* parental strains produced in a 24 h egg-laying in yeast for all the longevity trials. We also tested the same concentrations as in the toxicity/antitoxicity experiments. Lifespan assays were carried out at 25 °C, according to the procedure described by Fernandez-Bedmar, et al. [33]. Briefly, synchronised 72 ± 12 h old transheterozygous larvae were washed in distilled water, collected and transferred in groups of 100 individuals into test vials containing 0.85 g *Drosophila* Instant Medium and 4 mL of the different concentrations of the compound to be assayed. Emerged adults from pupae were collected under CO_2_ anaesthesia and placed in groups of 25 individuals of the same sex into sterile vials containing 0.21 g *Drosophila* Instant Medium and 1 mL of different concentrations of CAR. Flies were chronically treated during all their life. The number of survivors was determined twice a week. The statistical treatment of survival data was performed with the SPSS 19.0 (SPSS, Inc., Chicago, IL, USA) statistical software, using the Kaplan–Meier test. The significance of the curves was determined using the Log-Rank method (Mantel–Cox).

### 2.6. In Vitro Evaluation of Nutraceutical Potential

#### 2.6.1. Cytotoxicity Assay

The effect of the assayed compounds on cell viability was determined by the trypan blue exclusion test, according to our standard procedures [13]. HL-60 cells were placed in 96 well plates (2 × 10^4^ cells/mL) and cultured for 72 h and supplemented with the same concentrations of CAR from our toxicity assays. The wide range of tested concentrations was intended to estimate the cytotoxic inhibitory concentration 50 (IC_50_). After culture, cells were stained with a 1:1 volume ratio of trypan blue dye (T8154, Sigma-Aldrich, St. Louis, MO, USA) and counted in a Neubauer chamber at 100× magnification. The mean value of three independent assays of the alive treated cells was determined in order to obtain the tumoural growth inhibition curves. The standard error of the three replicas was calculated, and the curve given by the Excel Microsoft Office program was added. Finally, an estimation of inhibitory concentration 50 was calculated.

#### 2.6.2. DNA Fragmentation Status

The ability of our compound to induce DNA fragmentation was determined as described by Anter, et al. [34]. Briefly, 10^6^ HL-60 cells were co-cultured with five different concentrations of CAR (as selected in the toxicity assays) for 5 h. After treatment, genomic DNA was extracted using a commercial kit (Blood Genomic DNA Extraction Mini Spin Kit, Canvax Biotech, Cordoba, Spain). Subsequently, DNA was incubated overnight with RNAase at 37 °C and quantified in a spectrophotometer (Nanodrop_ND-1000, NanoDrop Technologies, Inc., Wilmington, DE, USA). Finally, 1200 ng DNA was electrophoresed in a 2% agarose gel for 40 min at 85 V, stained with GelRed and visualised under UV light. The apoptosis process can be detected by the appearance of internucleosomal DNA fragments that are multiple of 200 base pairs.

#### 2.6.3. Clastogenicity: SCGE (Comet Assay)

DNA integrity was assayed by SCGE as described by Mateo-Fernández, et al. [15], with minor modifications. HL-60 cells (5 × 10^5^) in exponential growing phase were incubated in 1.5 mL of culture medium supplemented with different CAR (0.03, 0.12 and 0.25 mg/mL) concentrations for 5 h. After treatment, cells were washed twice and adjusted to 6.25 × 10^5^ cells/mL in PBS. Electrophoresis gels were prepared, pouring a 1:4 dilution (cells in liquid low-melting-point agarose at 40 °C, A4018, Sigma-Aldrich, St. Louis, MO, USA) into slides. Gels were covered with a coverslip and allowed to solidify at room temperature (RT) for 15 min. Once the slides solidified, the coverslips were carefully removed and slides were bathed in freshly prepared lysing solution (2.5 M NaCl, 100 mM Na-EDTA, 10 mM Tris, 250 mM NaOH, 10% DMSO and 1% Triton X-100; pH 13) for 1 h at 4 °C. Thereafter, slides were equilibrated in alkaline electrophoresis buffer (300 mM NaOH and 1 mM Na-EDTA, pH 13) for 20–30 min at 4 °C. Once equilibrated, the slides underwent electrophoresis (12 V, 400 mA for 8 min) in the dark and were immediately neutralised in cold neutral solution (0.4 M Tris-HCl buffer, pH 7.5) for 5 min. Finally, slides were dried overnight at RT in the dark. Gels were stained with 7 µL propidium iodide and photographed in a Leica DM2500 microscope at 400× magnification (Leica Microsystems GmbH, Wetzlar, Germany). At least 100 single cells from each treatment were analysed, using the Open Comet software [35]. The Tail Moment (TM) data were analysed, applying a one-way ANOVA and post hoc Tukey’s test with SPSS Statistics for Windows, Version 19.0 (IBM Corporation, Armonk, NY, USA), to determine the effect of the tested compound on HL-60 cell DNA integrity.

#### 2.6.4. Methylation Status of HL-60 Cells

The methylation status assay was performed as it was described previously by Merinas-Amo, et al. [14]. Briefly, HL-60 cells were treated with different concentrations of CAR (0.12 mg/mL and 4 mg/mL) for 5 h. Then, DNA was extracted similarly to previously described DNA fragmentation assay. After that, the DNA was converted with bisulphite (EZ DNA Methylation-Gold Kit, Zymo Research, Irvine, CA, USA). Bisulphite-modified DNA was used for fluorescence-based real-time quantitative Methylation-Specific PCR (qMSP), using 5 µM of each forward and reverse primer (Isogen Life Science B.V., Utrecht, The Netherlands), 2 µL of iTaq Universal SYBR Green Supermix (Bio-Rad Laboratories, Inc., Hercules, CA, USA, it contains antibody-mediated hot-start iTaq DNA polymerase, dNTPs, MgCl_2_, SYBR Green Dye, enhancers, stabilizers, and a blend of passive reference dyes including ROX and fluorescein) and 25 ng of bisulphite converted genomic DNA. PCR conditions included initial denaturalisation at 95 °C for 3 min and amplification, which consisted of 45 cycles at 95 °C for 10 s, 60 °C for 15 s and 72 °C for 15 s, taking picture at the end of each elongation cycle.

After that, melting curve was determined, increasing 0.5 °C each 0.05 s from 60 °C to 95 °C and taking pictures. qMSP was carried out in 48 well plates in MiniOpticon Real-Time PCR System (MJ Mini Personal Thermal Cycler, Bio-Rad Laboratories, Inc., Hercules, CA, USA) and were analysed by Bio-Rad CFX Manager 3.1 software. The housekeeping Alu-C4 was used as a reference to correct for total DNA input. Alu-C4 and the target repetitive elements Alu M1, LINE-1 and Sat-α were obtained from Isogen Life Science B.V. (Utrecht, The Netherlands), and their sequences are shown in Table 1. Each sample was analysed in triplicate. The results of each CT (cycle number at which the amplification curves cross the threshold value) were obtained from each qMSP. Data were normalised with the housekeeping Alu-C4, using the Nikolaidis, et al. [36] and Liloglou, et al. [37] comparative CT method (ΔΔCT). One-way ANOVA and post hoc Tukey’s test were used to evaluate the differences between the tested compound, repetitive elements and concentrations.

## 3. Results

### 3.1. In Vivo Assays

Table 2 shows the toxicity and antitoxicity results obtained in this study. These results revealed that although CAR was significantly toxic at every tested concentration, the LD_50_ was not reached in any concentration. According to antitoxicity assay, antioxidant properties were not found in any tested concentrations but, contrarily, the highest concentration of CAR, which was even more toxic than both the positive controls in *D. melanogaster*.

Genotoxicity and Antigenotoxicity assays of CAR are shown in Table 3. The concurrent positive control showed significant differences with respect to the negative control using the Kastembaum–Bowman statistical test, providing a mutation rate per wing of 0.425 against 0.195, respectively. This result proves the accuracy of the assay. As regards genotoxicity, CAR showed inconclusive results, and it was solved by applying Mann–Whitney test, which demonstrated CAR was not a genotoxic compound. According to antigenotoxicity assay, combined treatments of CAR and hydrogen peroxide showed positive (*) results, which means that there were significant differences between CAR and the positive control. The IP was calculated since positive results were found. The IPs obtained in lowest and highest concentrations of CAR were 61% and 79.5%.

**Figure 1:**
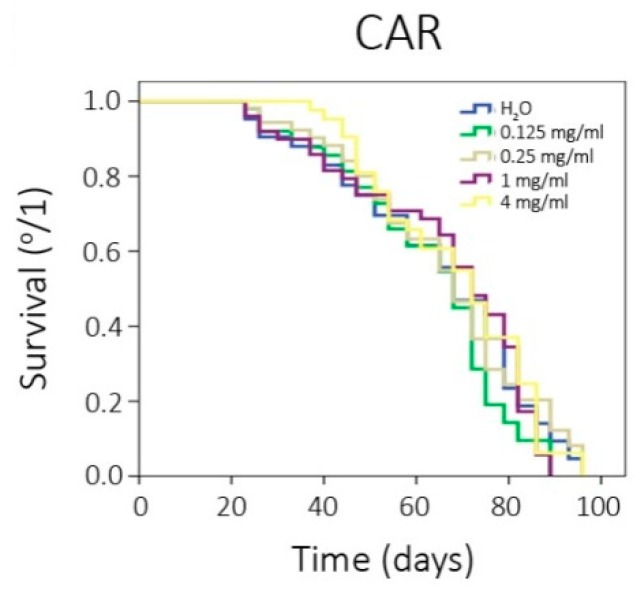
Survival curves obtained from log-rank test.

Table 4 shows the lifespan and healthspan results, which reported that CAR does not exert any significant effect on *Drosophila* lifespan and healthspan, based on the survival curves as they are depicted in Figure 1.

### 3.2. In vitro Assays

**Figure 2:**
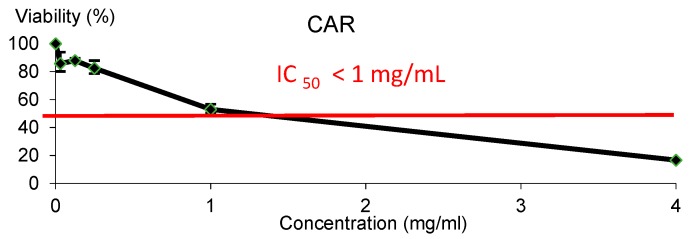
Viability of HL-60 cells treated with CAR for 72 h. Each point represents the percentage of viability with respect to the mean control ± SD of three independent experiments.

CAR showed cytotoxic effect against HL-60 leukaemia cell line, as it is shown in Figure 2. The IC_50_ was reached roughly at 1 mg/mL CAR.

**Figure 3:**
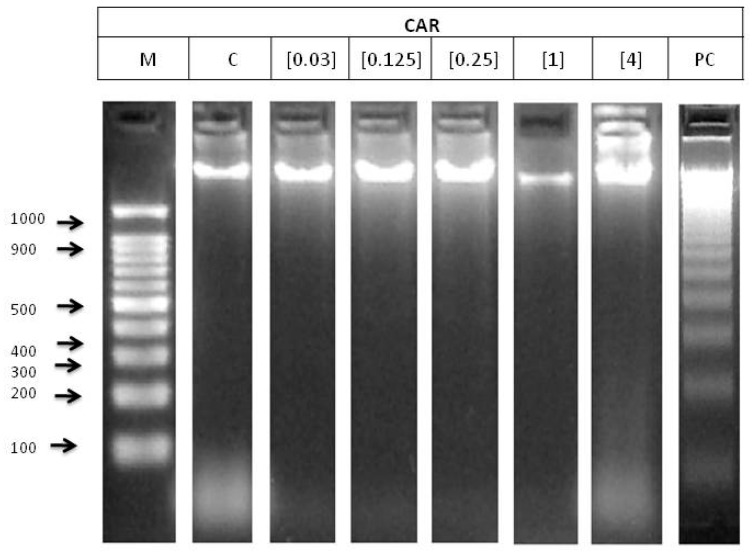
Internucleosomal DNA fragmentation after 5 h of HL-60 cells treated with CAR. Letters M and C mean weight size marker and negative control (RPMI), respectively, and lyophilised blond beer (62.5 mg/mL) has been used as a routine positive control (PC).

The electrophoresis of genomic DNA integrity of HL-60 cells treated with different concentrations of CAR is shown in Figure 3. No DNA damage was observed at any CAR concentrations.

**Figure 4:**
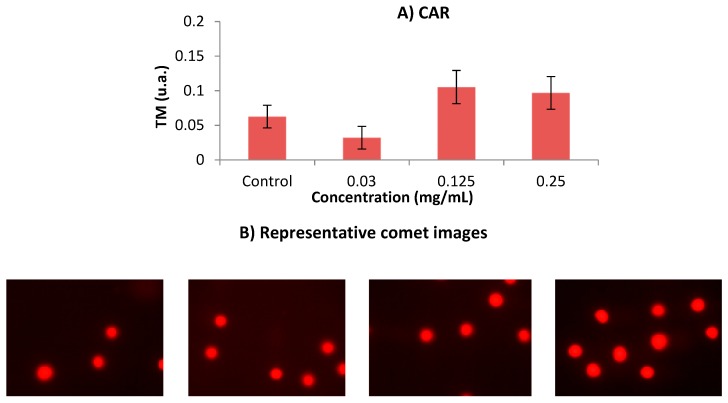
(**A**) Alkaline comet assay (pH > 13) of HL-60 cells after 5 h treatment with different concentrations of CAR. DNA migration is reported as mean TM. The plot shows mean TM values and standard errors. Statistical differences were analysed, applying one-way ANOVA and post hoc Tukey’s test. (**B**) Representative images of each dose group from comet assay are shown following the order described in Figure 4A (control, 0.03 mg/mL, 0.125 mg/mL and 0.25 mg/mL, respectively). TM: Tail Moment.

Figure 4 shows the results obtained in the single cell gel electrophoresis test or comet assay. According to this assay, CAR did not induce damage in human leukaemia HL-60 cell line at any tested concentration. The concentrations used in this SCGE assay were determined according to the results obtained in the previous cytotoxicity assay.

**Figure 5:**
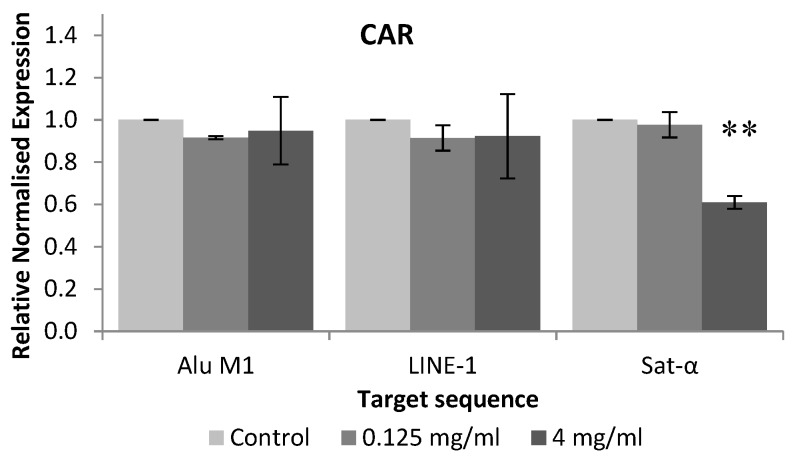
Relative normalised expression data of each repetitive element. Asterisk mark (*) is associated with different means, applying One-Way Anova test and post hoc Tuckey’s test.

Figure 5 shows the relative normalised methylation status (RMS) of the three repetitive sequences (LINE-1, Alu M1 and Sat-α) in HL-60 cell line treated with the tested compound. CAR hypomethylated Sat-α repetitive element of HL-60 cell line when these human leukaemia cells were treated with 4 mg/mL.

## 4. Discussion

### 4.1. Food Safety Assays: Toxicity and Genotoxicity

Food additives are still considered a big deal as many of their functions remain unknown. Precisely due to food colourants being massively used, an evaluation of their effect on public health should be needed [40]. Knowing that one third of human cancers are related to diet, much research is focused on small molecules added to food. This apparently easy concept became complicated as many compounds exert a dual, positive/negative effect that strongly depends on the dose [2]. The present work evaluates the safety of the caramel colour E150D (CAR) in in vivo toxicity and genotoxicity assays using the eukaryotic *Drosophila* model for the first time.

Our toxicity results revealed that although CAR was significantly toxic at every tested concentration, the LD_50_ was not reached in any concentrations. Therefore, CAR was not a toxic substance. This result did not agree with MacKenzie, et al. [41], who affirmed that caramel is not toxic. The toxicity of CAR could be caused by the 4-methyl imidazole (4-MeI) presence that has been described as a neurotoxic agent able to inhibit P450 cytochrome in the human liver [42,43] and even induced alveolar/bronchiolar adenoma and carcinoma in mice [44]. Anyway, The LD_50_ of CAR was not found at any concentrations, therefore both substances could be considered as nontoxic, being related to the report of MacKenzie, et al. [41].

Genotoxicity assays of tested compound showed inconclusive results, which were solved by applying Mann–Whitney test, which demonstrated that any of tested concentrations were genotoxic. This result agrees with the studies carried out by Brusick, et al. [45], who did not find evidence of genotoxicity in the *Salmonella* plate incorporation test using 5 standard strains or in the *Saccharomyces cerevisiae* gene conversion assay. The lack of genotoxicity was also demonstrated by Norizadeh Tazehkand, et al. [46], treating mice with 4-MeI. Colour caramel III was administrated to human males, and no significant differences were found in mean blood lymphocyte numbers compared to the respective control. The results supported the conclusion that this colourant does not pose a genotoxic hazard to humans [47] and CAR may provide nutraceutical potential.

The lack of genotoxicity observed in *Drosophila* for all CAR concentrations confirmed their safety. We hypothesised that the toxicity observed in our compound may either be induced by a different pathway than the genotoxic one, or it may be affecting different genes to those used in this assay.

### 4.2. Nutraceutical Potential Assays

Nutraceuticals and functional foods have become key issues in eating habits, nutrition and diets. The nutraceutical potential of food is recognised as an important domain of research [48]. The present study performed an evaluation of the nutraceutical potential of CAR by carrying out in vivo antitoxicity and lifespan assays as well as in vitro cytotoxicity, internucleosomal fragmentation, single and double DNA strands breaks and modulation of methylation patterns in the HL-60 leukaemia cells model.

### 4.3. In vivo Assays

In vivo antitoxicology assays have been performed through time in order to ascertain the health-promoting properties of the tested compounds. *D. melanogaster* model is increasingly used to study life extension, since there is a high homology between invertebrate and human genes involved in the aging process [17,49].

Our antitoxicity results demonstrated that CAR did not possess antioxidant effects in any tested concentrations since it was not able to revert the damage caused by hydrogen peroxide in *D. melanogaster*, except for the highest concentration of CAR, which was even more toxic than the positive control. According to antigenotoxicity assay, combined treatments of CAR and hydrogen peroxide showed positive (*) results, which means that there were significant differences between CAR and positive control, inhibiting the effect of the genotoxine in 61% and 79.5% for the lowest and the highest concentrations, respectively.

Tsai, et al. [50] concluded that CAR was overall antioxidant and this capacity depended on the colour of the caramel, and the brownest the additive the more antioxidant it is. Sengar and Sharma [10] reported a low antioxidant activity of CAR in a review. 4-MeI was demonstrated to be antigenotoxic in mice [46]. However, most of the literature regarding caramel focused on the identification of the caramelisation products. Therefore, more research is needed to evaluate the antioxidant and antigenotoxic properties of CAR as it is consumed.

The lifespan and healthspan results reported that CAR does not exert any significant effect on *Drosophila*’s lifespan and healthspan. As far as it is known, there is not any scientific information about CAR evaluating the aging and lifespan, only based on 4-MeI, which is one of the main components of CAR. 4-MeI was used in a chronic treatment conducted in rats. No observable adverse effects were found [41,47], being our results consistent with those obtained.

### 4.4. In vitro Assays

The in vitro evaluation of the anti-cancer properties of nutraceutical compounds is the first step of a large pathway to obtain suitable conclusions to be extrapolated to humans. The aim of the present trial was to determine the potential chemopreventive and genotoxic effect of CAR on a human cancer cell model (HL-60 cell line), performing cytotoxicity, DNA fragmentation, SCGE and epigenetic modulation assays.

CAR showed cytotoxic effect against HL-60 leukaemia cell line, and the IC_50_ was reached roughly at 1 mg/mL for CAR inducing cell death in HL-60 cell line. It seems to be the first attempt on ascertaining the chemopreventive potential of CAR. Our findings agreed in some extent with the cytotoxicity activity observed in lymphocytes induced by caramel colour additive [41]. Therefore, further research studies are needed to ascertain the chemopreventive potential of CAR.

Clastogenicity is involved in a process of DNA damage. DNA fragmentation test and comet assay were conducted in order to examine the clastogenic potential of CAR on HL-60 promyelocytic cell line. The degradation of genomic DNA into internucleosomal fragments was proposed as a major mechanism affecting cancer cell apoptosis. The typical ladder pattern has not been shown by any of the tested concentrations, thus they are not able to induce apoptosis (Figure 3). This result is not consistent with the reduction of the number of the mice follicles and oocytes observed by Suocheng, et al. [51], who concluded that this decrease could be due to apoptotic mechanisms. However, the cells used in this research differed from the used one in the present study. National Toxicology Progam (NTP) concluded there was no evidence of carcinogenic activity of 4-MeI in rats, but this compound should be better named as “some evidence”, according the information found in previous studies [47].

Alkaline SCGE is performed in order to detect DNA damage [52], which is widely used to determine whether cells are undergoing apoptotic and/or necrotic pathways [53]. It is assumed that apoptosis occurs when treatments induce a TM > 30 (hedgehog pattern), whereas control cells remain lower than 2 (no tails). On the contrary, necrosis shows a short comet-tail pattern since the majority of the damaged DNA remains in the comet head [54]. Our result showed that CAR did not exhibit clastogenic activity, since TM values of all assayed concentrations remained in TM values lower than 1, as it is depicted in Figure 4. The concentrations used in this SCGE assay were determined according to the results obtained in the previous cytotoxicity assay. This finding means that CAR can be regarded as untreated cells (class 0) from the five TM classes proposed by Fabiani, et al. [55].

Clastogenic activity in CHO cells was induced when exposed to caramel colour [56]. These results are not in agreement with our findings, although the experimental models were different. The lack of in vitro genetic damage could be due to the fact that the assessed concentrations in comet assay were the three lowest ones, which are the less cytotoxic being the cell viability is roughly 80%. Furthermore, the results obtained in comet assay are congruent with those obtained in our DNA fragmentation test.

Despite the cytotoxic activity shown by CAR, internucleosomal DNA fragmentation and DNA damage at the assayed concentrations were not induced, thus the cell death was not due to proapoptotic mechanisms in our HL-60 model.

As for epigenetics, the genome is globally hypomethylated in cancer cells, inducing transposable element activity and thus triggering genome instability [57]. On the other hand, it is known that the silencing of tumour suppressor genes is closely associated with hypermethylation [58]. Repetitive elements are highly methylated in somatic normal cells, contributing to a global genomic hypermethylation [38], suppressing the transposable activity of repetitive elements. Three different repetitive elements: LINE-1, Alu-M4 and Sat-α were studied. Long interspersed nuclear elements (LINE) are abundant retrotransposons, and representing LINE-1 about 17% of the human genome, with a nonrandom distribution by accumulating primarily in G-positive bands, which are AT-rich regions of chromosomes [59]. LINE-1 elements are also accumulated in regions of low recombination rate, mainly in X-chromosome [60]. Alu elements belong to the SINE family (Short Interspersed Nuclear Elements), being the most abundant (accounting about 10% of the whole human genome [38] and predominantly present in noncoding and GC-rich regions [59,61]. Sat-α (Satellite alpha DNA) repeats are composed of tandem repeats of 170 bp DNA sequences, are AT-rich regions and represent the main DNA component of every human centromere, constituting about 5% of total human DNA [59,62]. Therefore, examination of the methylation status of LINE-1, Alu and Sat-α genomic regions has served as an approach for measuring global methylation levels since 32 % of the human genome has been evaluated [63].

To our knowledge, this is the first attempt at evaluating the ability of CAR for modulating the epigenome, thus there is not any information related to this assay using CAR on the scientific database. Our results showed that CAR hypomethylated Sat-α repetitive element of HL-60 cell line when these human leukaemia cells were treated with 4 mg/mL. In addition, it has been demonstrated that the expression of satellite sequences is associated with a hypomethylation, triggering cancer cells. Therefore, methylation process in satellite sequences is a potential mechanism for silencing its satellite expression in transformed cells [64], which is not induced by CAR.

Some recent human therapies against cancer are based on hypomethylating agents, since this activity is highly related to gene silencing, thus this fact could activate tumour suppressor genes and be a positive highlight. Despite this fact, it is not clear its benefit on human therapies, and many more research studies should be performed [65].

Further research using normal human cell line should be taken into account, to be compared to our carcinogenic cells once CAR is recommended to be monitored and reduced in soft drinks [66].

## 5. Conclusions

In conclusion, the food safety of CAR was demonstrated, since LD50 was not reached in toxicity assay and any of the tested concentrations induced mutation rates higher than that of the concurrent control in *D. melanogaster*. On the other hand, CAR protected DNA from oxidative stress provided by hydrogen peroxide in *D. melanogaster,* according to antigenotoxiciy assay. In addition, CAR was a first-step chemopreventive compound but it did not induce clastogenicity in human leukaemia cells. CAR modified the methylation status of HL-60 cell line, although much more information about the mechanisms of gene therapies related to epigenetic modulation by food is necessary.

## Figures and Tables

**Table 1:** Primers information [38].

Primer	Forward Primer Sequence 5′ to 3′ (N)	Reverse Primer Sequence 5′ to 3′ (N)
ALU-C4	GGTTAGGTATAGTGGTTTATATTTGTAATTTTAGTA (-36)	ATTAACTAAACTAATCTTAAACTCCTAACCTCA (-33)
ALU-M1	ATTATGTTAGTTAGGATGGTTTCGATTTT (-29)	CAATCGACCGAACGCGA (-17)
LINE-1-M1	GGACGTATTTGGAAAATCGGG (-21)	AATCTCGCGATACGCCGTT (-19)
SAT-α-M1	TGATGGAGTATTTTTAAAATATACGTTTTGTAGT (-34)	AATTCTAAAAATATTCCTCTTCAATTACGTAAA (-33)

ALU (Short interspersed nuclear element –SINE- Alu-C4 sequence). LINE (Long Interspersed Nuclear Element M1). Sat-α (Satellite alpha DNA).

**Table 2:** Toxicity and antitoxicity levels of CAR in *D. melanogaster*.

CAR (mg/mL)	Survival (%)	
	Simple Treatment ^1^	Combined Treatment ^2^
**0**	100	100
**H_2_O_2_**	-	62.64
**0.03**	61 *^,3^	62
**0.125**	65.7 *	54.02
**0.25**	64.3 *	53
**1**	61.32 *	54.02
**4**	63 *	23 *^,4^

^1^ Data are expressed as percentage of survival adults with respect to 300 untreated 72 h old larvae from three independent experiments. ^2^ Combined treatments using standard medium and 0.15 M hydrogen peroxide. ^3^ Asterisks (*) indicate significant differences (one tail) with respect to the untreated control group and ^4^ the hydrogen peroxide control group: * Chi-square value higher than 5.02 [15]. CAR: caramel colour E150d-class IV.

**Table 3:** Genotoxicity and Antigenotoxicity assays of CAR in *D. melanogaster*.

Clones per Wings (Number of Spots)							
Compound	Wings Number	Small Single Spots (1–2 Cells) *m* = 2	Large Simple Spots (>2 Cells) *m* = 5	Twin Spots *m* = 5	Total Spots *m* = 2	Mann–Whitney Test	IP (%)
**H2O**	41	0.147 (6)	0.048 (2)	0	0.195 (8)		
**H2O2 (0.15 M)**	40	0.375 (15)	0.05 (2)	0	0.425 (17) +		
**SIMPLE TREATMENT**							
**CAR (mg/mL) [0.125]** **[4]**	40	0.25 (10)	0.125 (5)	0	0.375 (15) i	Λ	
	42	0.166 (7)	0.095 (4)	0.024 (1)	0.286 (12) i	Λ	
**COMBINED TREATMENT**							
**CAR (mg/mL)** **[0.125]** **[4]**	42	0.166 (7)	0	0	0.166 (7) *		61
	46	0.065 (3)	0.02 (1)	0	0.087 (4) *		79.5

Statistical diagnosis according to Frei and Wurgler [39]: + (positive), − (negative) and i (inconclusive) vs. negative control; * (positive), Δ (negative) and β (inconclusive) vs. respective positive control; m: multiplication factor. Kastenbaum–Bowman Test without Bonferroni correction, probability levels: α = β = 0.05. No. of spots in parentheses. Mann-Whitney test was used when appropriate to resolve inconclusive results. Lambda (Λ) symbol mean that there were not significant differences with respect to the negative control when Mann-Whitney test is applied. Inhibition percentage values were included when appropriate.

**Table 4:** Effects of CAR treatments on the *Drosophila melanogaster* mean lifespan and healthspan.

CAR (mg/mL)	Mean Lifespan (Days)	Mean Lifespan Difference (%) ^a^	Healthspan (80th Percentile) (Days)	Healthspan Difference (%) ^a^
Control	64 ± 3.16	0	31.21 ± 2.37	0
0.125	59.65 ± 2.4	−6.8	31.03 ± 2.12	−0.5
0.25	60.83 ± 2.73	−4.9	33.68 ± 2.44	7.6
1	62.8 ± 2.78	−1.9	30.88 ± 2.1	−1.1
4	59 ± 3.35	−7.9	37.54 ± 4	20.28

^a^ The difference was calculated by comparing treated flies with the concurrent water control. Positive numbers indicate lifespan increase, and negative numbers indicate lifespan decrease. Data are expressed as mean value ± SE.
